# Application of a combined cancellous lag screw enhances the stability of locking plate fixation of osteoporotic lateral tibial plateau fracture by providing interfragmentary compression force

**DOI:** 10.1186/s13018-024-04564-8

**Published:** 2024-02-14

**Authors:** Jiang Jiang, Daqiang Xu, Zhenhua Ji, Fei Wang, Rui Jia, Jun Wang, Hong Hong, Hongtao Zhang, Jianyi Li

**Affiliations:** 1 Department of Anatomy, Guangdong Provincial Key Laboratory of Digital Medicine and Biomechanics, Guangdong Engineering Research Center for Translation of Medical 3D Printing Application, National Virtual and Reality Experimental Education Center for Medical Morphology, School of Basic Medical Sciences, Southern Medical University, No. 1023, South Shatai Road, Baiyun District, Guangzhou, 510515 Guangdong China; 2 Department of Orthopedics, Affiliated Hospital 6 of Nantong University, Yancheng Third People’s Hospital, Yancheng, China; 3 Department of Rehabilitation Medicine, Shandong Public Health Clinical Center, Shandong University, Jinan, China; 4 Department of Rehabilitation Medicine, Guangdong Provincial People’s Hospital (Guangdong Academy of Medical Sciences), Southern Medical University, Guangzhou, China; 5 Zhongshan Torch Development Zone People’s Hospital, No.123, Yixian Road, Torch Development District, Zhongshan, 528437 Guangdong China

**Keywords:** Combined cancellous lag screw, Locking plate, Osteoporotic lateral tibial plateau fracture, Stable fixation, Interfragmentary compression force

## Abstract

**Background:**

Insufficient interfragmentary compression force (IFCF) frequently leads to unstable fixation of osteoporotic lateral tibial plateau fractures (OLTPFs). A combined cancellous lag screw (CCLS) enhances IFCF; however, its effect on OLTPF fixation stability remains unclear. Therefore, we investigated the effect of CCLS on OLTPF stability using locking plate fixation (LPF).

**Materials and methods:**

Twelve synthetic osteoporotic tibial bones were used to simulate OLTPFs, which were fixed using LPF, LPF-AO cancellous lag screws (LPF-AOCLS), and LPF-CCLS. Subsequently, 10,000 cyclic loadings from 30 to 400 N were performed. The initial axial stiffness (IAS), maximal axial micromotion of the lateral fragment (MAM-LF) measured every 1000 cycles, and failure load after 10,000 cycles were tested. The same three fixations for OLTPF were simulated using finite element analysis (FEA). IFCFs of 0, 225, and 300 N were applied to the LPF, LPF-AOCLS, and LPF-CCLS, respectively, with a 1000-N axial compressive force. The MAM-LF, peak von Mises stress (VMS), peak equivalent elastic strain of the lateral fragment (EES-LF), and nodes of EES-LF > 2% (considered bone destruction) were calculated.

**Results:**

Biomechanical tests revealed the LPF-AOCLS and LPF-CCLS groups to be superior to the LPF group in terms of the IAS, MAM-LF, and failure load (all *p* < 0.05). FEA revealed that the MAM-LF, peak VMS, peak EES-LF, and nodes with EES-LF > 2% in the LPF were higher than those in the LPF-AOCLS and LPF-CCLS.

**Conclusion:**

IFCF was shown to enhance the stability of OLTPFs using LPF. Considering overscrewing, CCLS is preferably recommended, although there were no significant differences between CCLS and AOCLS.

## Background

Osteoporotic tibial plateau fracture (TPF) is prevalent among elderly populations [1]. Due to specific knee geometry and tibiofemoral joint force, over 60% of osteoporotic TPFs occur in the lateral column [2]. The prevalence of osteoporotic lateral TPF (OLTPF) gradually increases with age [3], and this injury can have a catastrophic effect on the health of the elderly.

Stable fixation for OLTPF is critical for accelerating recovery in elderly patients and avoiding complications related to long-term bed rest [4, 5]. Among the various available fixation methods, locking plate (LP) fixation (LPF) is the most widely used for OLTPF [6, 7]. However, 11% of patients with LP-fixed lateral TPF experience lateral platform collapse [8, 9]. Gardner et al. found that locking screw-cutting in the cancellous epiphyseal area was an important contributing factor, possibly due to the increasing shear stresses at the locking screw-bone interface [10]. Therefore, additional lag screws were applied to increase the interfragmentary compression force (IFCF), thereby reducing the shear stresses at the locking screw-bone interface and ultimately enhancing the stability of the LPF of the lateral TPF [11, 12]. However, due to severe bone mass reduction in the osteoporotic tibia, the commonly used cancellous lag screws are prone to overscrewing, resulting in a decrease in IFCF and a high risk of lateral platform collapse [13, 14].

The combined cancellous lag screw (CCLS) previously developed by our research team may offer promise for solving this issue [13]. The major improvement in this device is that the screw rod of the CCLS is divided into two parts and connected by fine threads. This allows the screwing angle range to be expanded through fine threads, enabling surgeons to accurately determine the time to stop screw insertion to obtain a greater IFCF and avoid overscrewing. Moreover, this device facilitates the avoidance of further cutting damage to the osteoporotic cancellous bone by screw threads, as it fastens fragments by shortening the screw length through fine threads. Based on these characteristics, CCLS obtained a 25% higher IFCF than AO cancellous lag screws (AOCLS) in osteoporotic bones [15]. However, no studies have investigated the effect of CCLS on the stability of OLTPF using LPF.

Consequently, this study aimed to investigate the effect of CCLS on the stability of OLTPF using biomechanical testing and finite element analysis (FEA). We hypothesized that the CCLS would effectively enhance the stability of the LPF of OLTPF by providing IFCF.

## Materials and methods

### Biomechanical testing

#### Materials

Twelve large fourth-generation osteoporotic synthetic left tibial bones (No. #3402 Sawbones, 0.16 g/cc, Pacific Research Laboratories, Vashon, WA, USA) were used in this study [16]. The synthetic tibial bones were transversely truncated 200 mm from the lateral plateau and fixed using a dental tray powder (polymethyl methacrylate, Shanghai New Century Dental Materials Co., Ltd, Shanghai, China).

#### Fracture models and test groups

**Fig. 1:**
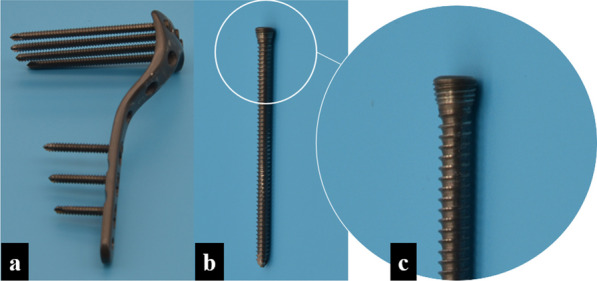
Locking plate system for biomechanical testing and modeling in our study. **a** Locking plate system; **b** Locking screw; **c** Head of the locking screw

A reproducible cut was mechanically created by the same surgeon who used a thin blade saw based on a single template to create a lateral tibial plateau fracture (Schatzker type I). Following anatomical reduction under direct vision, the synthetic tibial bones were randomly instrumented into three groups of four samples each:

- *LPF* A single lateral proximal tibia LP (left; thickness, 4.0 mm; length, 106 mm; Jiangsu Jinlu Medical Device, Inc., Zhangjiagang, China) was fixed with seven locking screws (4.0 mm diameter, Jiangsu Jinlu Medical Device, Inc., Zhangjiagang, China; Fig. 1). All locking screws were tightened with a torque of 4 Nm, and no IFCF was applied.
- *LPF with AOCLS (LPF-AOCLS)* An AOCLS (6.5S*65 mm; thread length, 18 mm; Changzhou Geasure Medical Apparatus and Instruments Co., Ltd., China) with a washer was used to fix the lateral fragment to the maximum IFCF perceived by the surgeon. An LP was then implanted with seven locking screws, and all locking screws were tightened with a torque of 4 Nm.
- *LPF with CCLS (LPF-CCLS)* A CCLS (6.5S*65 mm; thread length, 18 mm; Changzhou Geasure Medical Apparatus and Instruments Co., Ltd., China) with a washer was used to fix the lateral fragment. Rod length was reduced using a custom-designed locked screwdriver to obtain the maximum IFCF. Finally, the LP was implanted with seven locking screws, and all locking screws were tightened with a torque of 4 Nm.

#### Test procedure

**Fig. 2:**
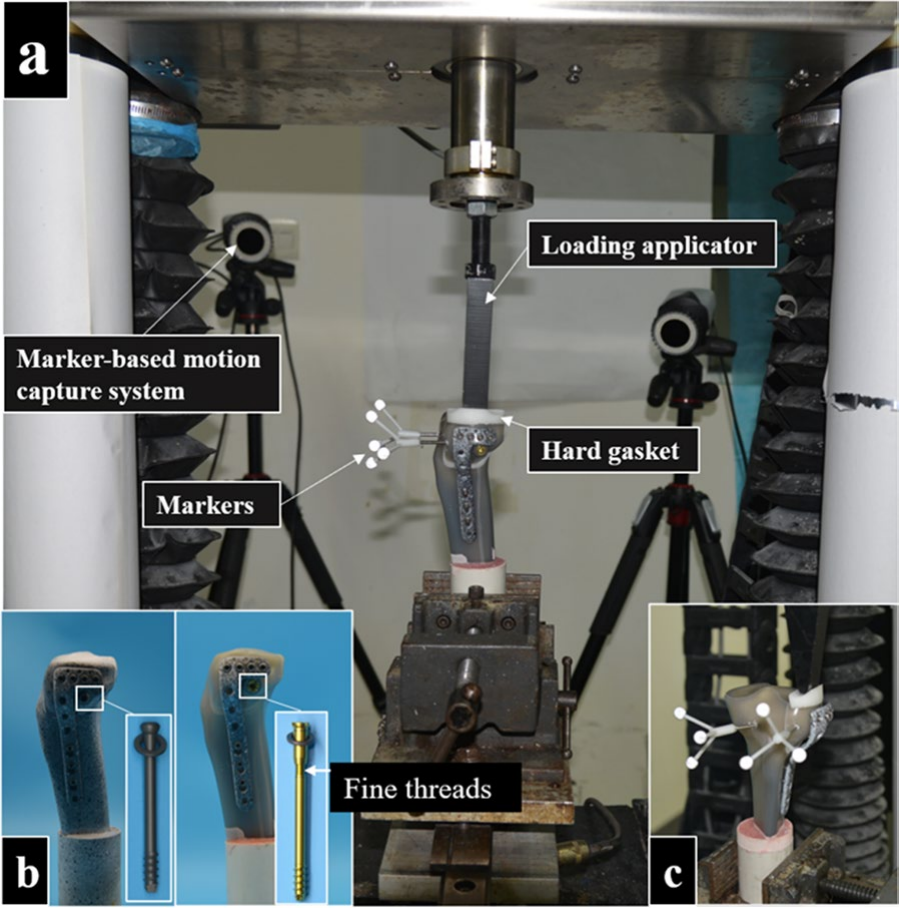
Preparation of samples and accessories. **a** Views of constructs mounted for testing; **b** Two different lag screws for osteoporotic lateral tibial plateau fracture (OLTPF) reduction; c) A hard gasket was matched between the upper surface of the lateral fragment and the loading applicator

All samples were subjected to compression loading to replicate the shearing forces on the tibial plateau during complete knee extension using a specially designed loading applicator. A hard gasket was attached to the upper surface of the lateral fragment, and a four-camera marker-based motion capture system (120 Hz, Qualysys AB, Gothenburg, Sweden) was used to obtain interfragmentary displacements. The mechanical properties of the samples were measured using a BOSE3510-AT testing machine (load measuring range, ± 7500 N, displacement measuring range, ± 25 mm, frequency range, 0.00001–100 Hz, Bose Corporation, Force Systems Group, Eden Prairie, MN, USA; Fig. 2).

After the models were created, the bones were subjected to 10,000 cyclic loadings with forces ranging from 30 to 400 N. The waveform of the cyclic loading was sinusoidal, with a frequency of 3 Hz. Fixation failure was defined as synthetic bone fracture, implant fracture, or disengagement of the bone–implant relationship. If the samples did not exhibit failure within 10,000 cycles, a load-to-failure test was performed at a loading speed of 5 mm/min until a displacement of 3 mm could be achieved.

The initial axial stiffness (IAS), maximal axial micromotion of the lateral fragment (MAM-LF) during cyclic loading, failure loads, and number of failure cycles (for structures that failed within 10,000 cycles) were assessed. IAS was defined as the force–displacement ratio at the third loading [17, 18]. The load that caused a 2-mm displacement was identified as the failure load.

### FEA

#### Experimental models

**Fig. 3:**
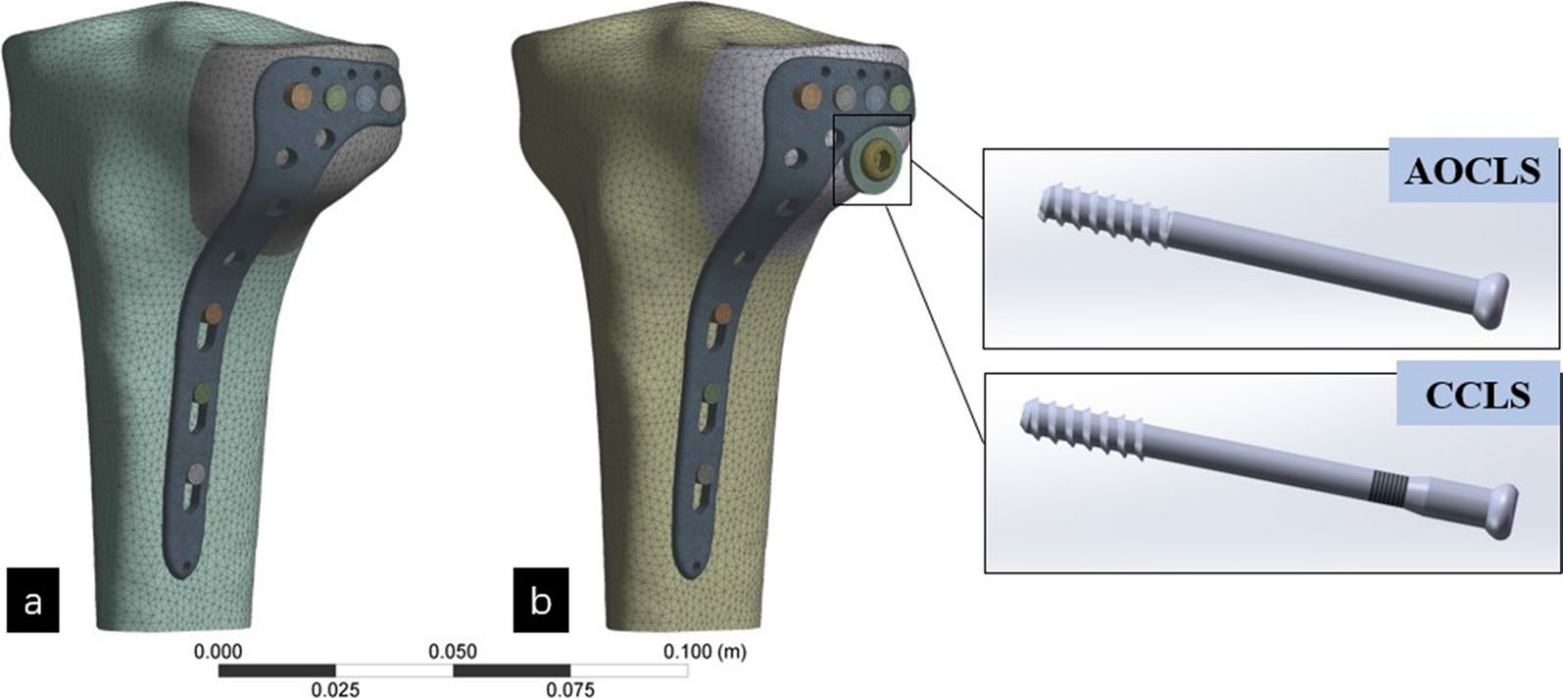
Three fixations of the finite element models: **a** Locking plate fixation (LPF); **b** LPF with AO cancellous lag screw (LPF-AOCLS), and LPF with combined cancellous lag screw (LPF-CCLS)

Experimental models, including the AOCLS, CCLS, and LP systems, were constructed based on the specifications provided by their manufacturers. The left proximal tibia model was generated using Mimics v19.0 software (Materialize Mimics, Leuven, Belgium) to create a three-dimensional reconstruction of computed tomography scan data obtained from a 27-year-old healthy volunteer (male; height, 174 cm; weight, 70 kg), from whom informed consent was obtained prior to data collection. Standard radiographs were performed to exclude lower-extremity fractures, abnormalities, and pathologic bone lesions. The scans were performed on a Philips Ingenuity 64 CT scanner. The scan range was from the anterior superior iliac spine to the ankle. The scan parameters were as follows: 140 kV; 350 mAs; slice thickness: 1 mm; scanning interval: 0.5 mm. The Digital Imaging and Communications in Medicine data of 1000 layers were copied and recorded. Ethical approval was granted by the Institutional Ethics Committee of our institution (Ethical Clearance Certificate No. 2022-01). Subsequently, the model was imported into SolidWorks (version 2017; Dassault Systèmes, Waltham, MA, USA) to create the lateral TPF (Schatzker I) and to virtually implant all devices into the fractured proximal tibia to simulate the three fixations: LPF, LPF-AOCLS, and LPF-CCLS (Fig. 3a, b). An appropriate position was maintained between the LP and tibia during the modeling to guarantee that all models maintained the precise relative positions of the LP and tibia in order to ensure accurate calculations. Once the relative positions of the LP and tibia were established, they were replicated and employed as the foundational model for subsequent models [19].

The assembled models were then submitted to ANSYS (version 17.0, ANSYS, Inc., Canonsburg, PA, USA) to mesh. Meshes were generated using Solid187 10-node tetrahedral elements consisting of 1,179,829, 1,297,907, and 1,287,355 nodes in the LPF, LPF-AOCLS, and LPF-CCLS models, respectively [20, 21]. The material properties were defined as homogeneous, isotropic, and linearly elastic (summarized in Table 1) [22, 23].

**Table 1:** Material properties of the FE models used in this study

Material	Young’s modulus, mPa	Poisson’s ratio	Material type
Cancellous bone	34	0.2	Osteoporotic cancellous bone
Cortical bone	8040	0.3	Osteoporotic cortical bone
Screws	110,000	0.3	Titanium alloy
Locking Plate	110,000	0.3	Titanium alloy

### Boundary and loading conditions

**Fig. 4:**
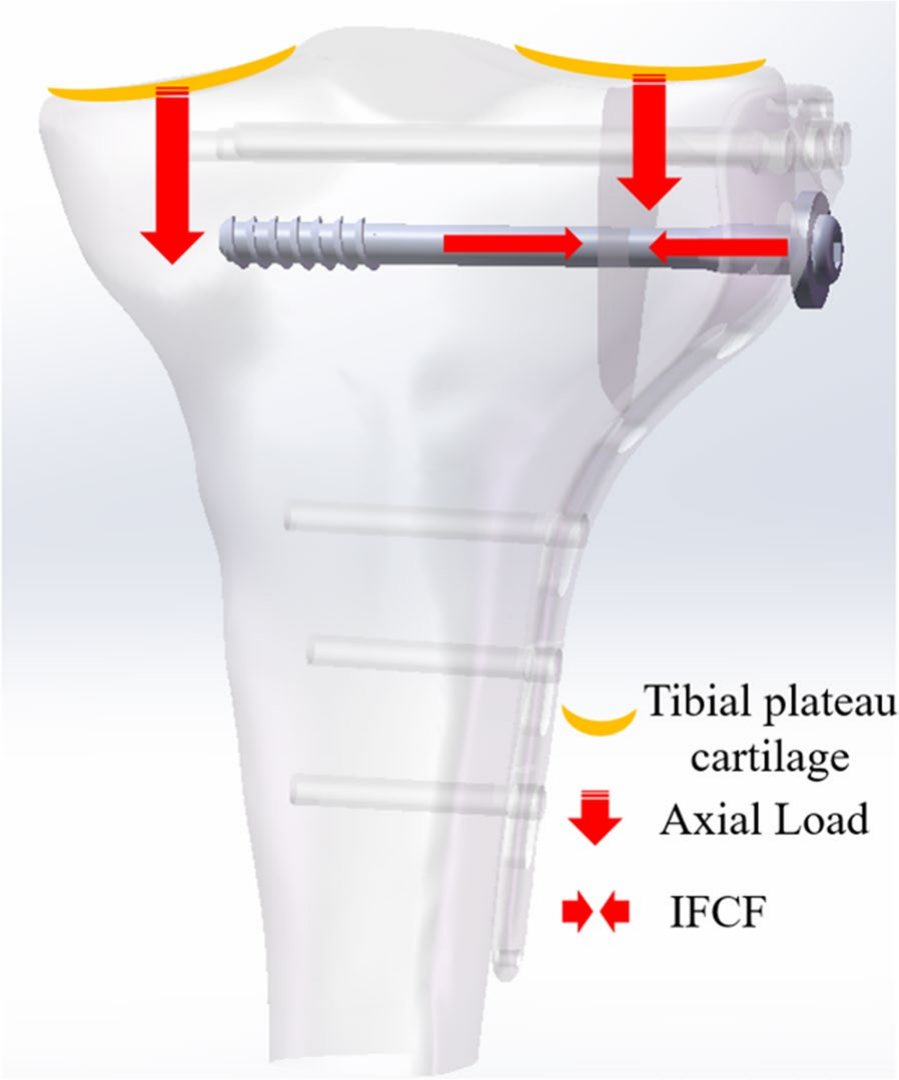
Depictions of the loads applied in the finite element models

According to clinical practice and previous studies [19, 21], the contact between the LP and screws was fully bound to imitate the locking mechanism. The screws were fully tied to the tibia, and a friction coefficient of 0.3 was used for the fragment–screws and fragment–tibia interactions. The degrees of freedom on the surface of the distal tibia were fully constrained. The distal ends of the tibial models were fully constrained as the boundary condition. IFCFs of 0, 225, and 300 N were then applied to the LPF, LPF-AOCLS, and LPF-CCLS, respectively [15]. An axial compressive force of 1000 N was applied to simulate the walking load in an adult patient. Of these, 60% of the selected force was attributed to the medial tibial plateau and 40% to the lateral tibial plateau (Fig. 4).

### Convergence analysis, model validation, and analysis

Convergence analysis of the meshes was performed to determine the appropriate mesh quality (convergence change rate < 2%) [24]. The MAM-LF, peak von Mises stress (VMS), and peak equivalent elastic strain of the lateral fragment (EES-LF) were then assessed. The MAM-LF was evaluated to validate the finite element (FE) model, which was compared using biomechanical tests. MAM-LF > 2 mm and EES-LF > 2% indicated failure displacement and bone destruction, respectively [25].

### Statistical analysis

Statistical analyses were performed using IBM SPSS (version 27.0; IBM Corp., Armonk, NY, USA). The Shapiro–Wilk normality test was performed to check for data normality. An analysis of variance was used to compare differences among the three groups, and Fisher’s Least Significant Difference test was used as a post hoc test.

## Results

### Biomechanical testing

**Fig. 5:**
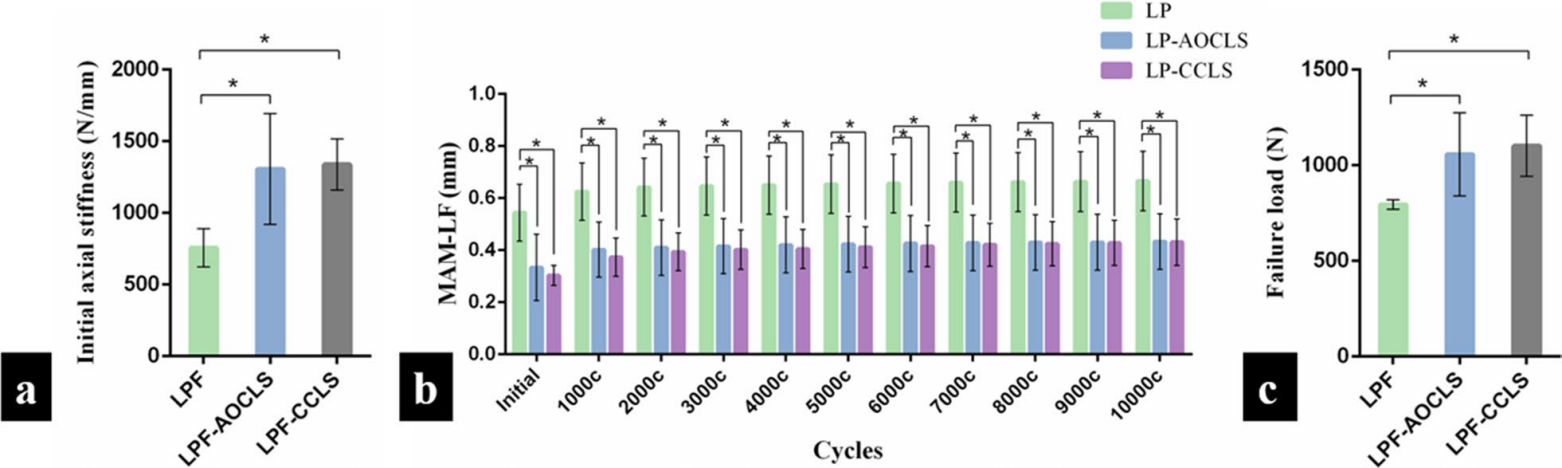
Results of biomechanical testing. **a** Initial axial stiffness (IAS): The locking plate fixation (LPF) group showed significant differences compared with the LPF-AO cancellous lag screw (AOCLS) and LPF-combined cancellous lag screw (CCLS) groups (*p* < 0.05); **b** Maximal axial micromotion of the lateral fragment (MAM-LF) in cyclic compression loading in every 1000 cycles: MAM-LF in the LPF group showed significant differences compared with that in the LPF-AOCLS and LPF-CCLS groups measured at every 1000 cycles (*p* < 0.05); **c** Failure load in the load-to-failure test: There was a significant difference in failure load when the LPF group was compared with the LPF-AOCLS and LPF-CCLS groups (*p* < 0.05). *p value < 0.05

The IAS test revealed a significant difference between the LPF group (754.54 ± 134.43 N/mm) and the LPF-AOCLS group (1,305.40 ± 386.71 N/mm). A significant difference was also found between the LPF and LPF-CCLS (1,336.893 ± 176.921 N/mm) groups (*p* < 0.05; Fig. 5a).

No fixation failure was observed in any samples during the loading cycles. The MAM-LF of the LPF group showed statistically significant differences compared to the LPF-AOCLS and LPF-CCLS groups when measured after every 1000 cycles (*p* < 0.05; Fig. 5b).

The load-to-failure test revealed statistically significant differences in failure load in the LPF group (794.848 ± 24.99 N), which were compared to those in the LPF-AOCLS (1,057.122 ± 216.84 N) and LPF-CCLS (1,101.470 ± 160.09 N) groups (*p* < 0.05; Fig. 5c).

### FEA

#### Convergence analysis and FE model validation

Mesh sizes of 2.0 mm for bones and 0.5 mm for implants were applied in this study according to the results of the mesh convergence analysis (Table 2). The MAM-LF in the FE model was 0.596 mm, similar to the results of the biomechanical test (0.5446 ± 0.1092 mm). The FEA model herein was, therefore, deemed valid.

**Table 2:** Mesh convergence analysis of the finite element models

Meshing schemes of the FE model	Scheme 1	Scheme 2	Scheme 3	Scheme 4	Scheme 5	Scheme 6
Bone mesh size (mm)	1.8	2	2.2	2.4	2.6	2.8
Implant mesh size (mm)	0.4	0.5	0.6	0.7	0.8	0.9
Number of elements (bone)	394,567	283,144	211,575	162,570	130,294	105,606
Number of elements (implants)	927,049	476,396	279,679	176,629	118,313	83,809
Analysis time (min)	113	90	42	44	29	15
Maximum von Mises stress of the bone (mPa)	13.521	12.314	12.47	12.154	12.585	13.44
von Mises stress change rate (bone)	− 9.80%	1.25%	− 2.60%	3.42%	6.36%	–
Maximum von Mises stress of the implants (mPa)	99.186	102.14	101.33	96.872	100.58	95.029
von Mises stress change rate (implants)	2.89%	− 0.80%	− 4.60%	3.69%	− 5.84%	–

### MAM-LF

**Fig. 6:**
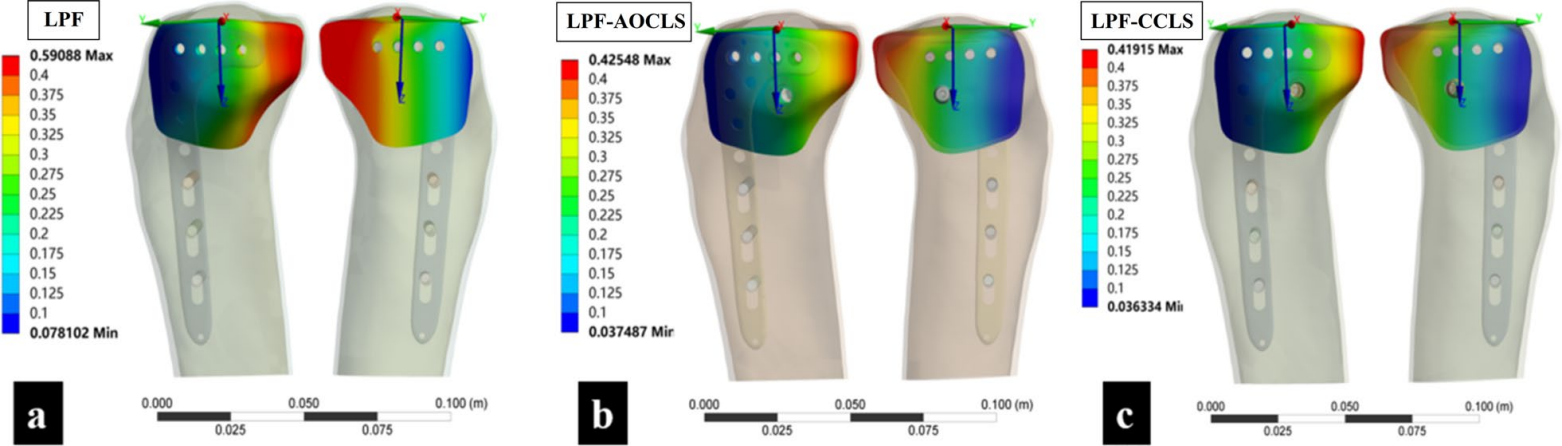
Comparison of the three fixation models of MAM-LF in the finite element analysis. **a** LPF model; **b** LPF-AOCLS model; **c** LPF-CCLS model

No failed displacements of the lateral fragments were observed in any model. The MAM-LFs were 0.5909, 0.4255, and 0.4192 mm for the LPF, LPF-AOCLS, and LPF-CCLS models, respectively (Fig. 6).

### Peak VMS of implants

**Fig. 7:**
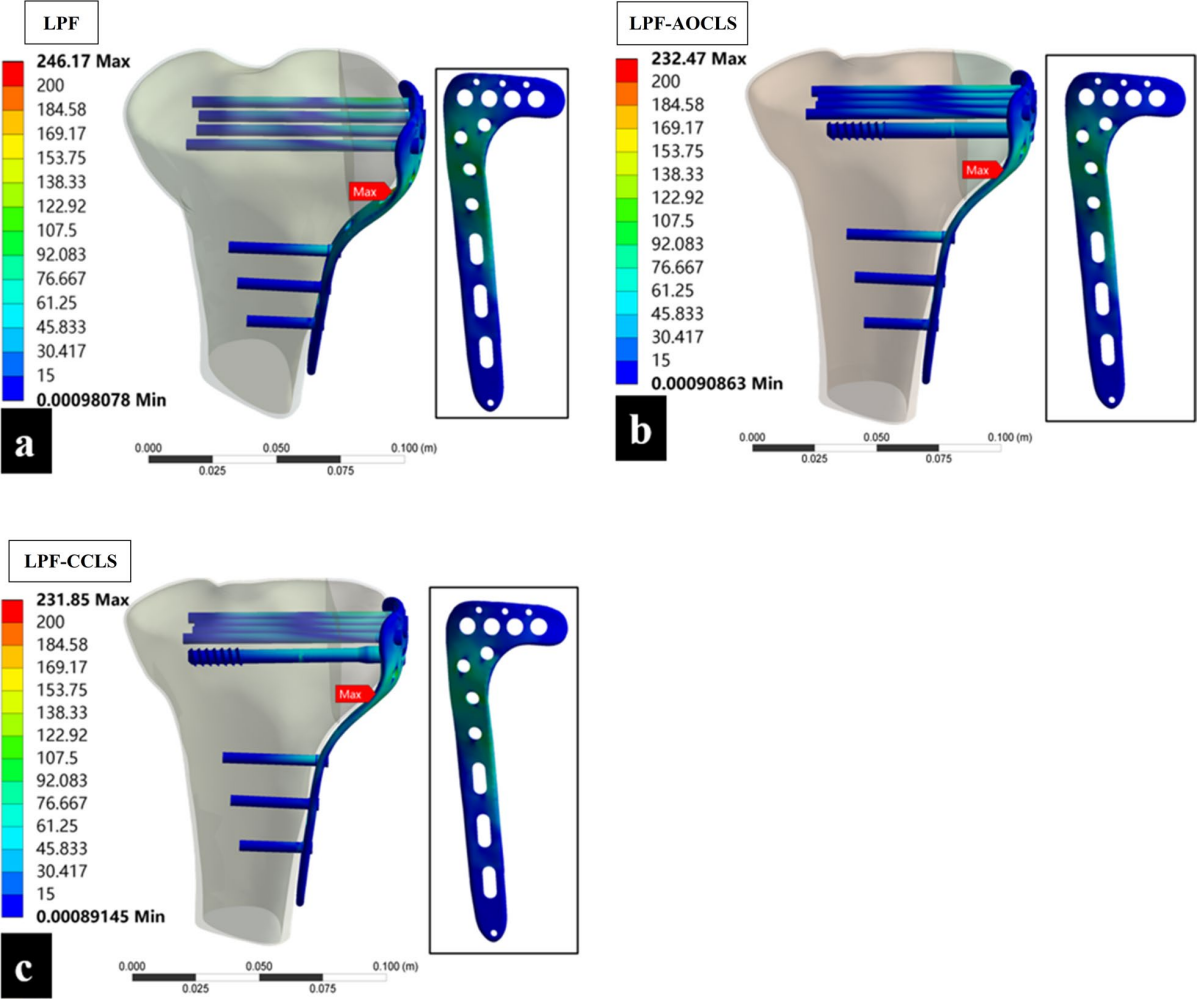
Comparison of implants of peak von Mises Stress (VMS) of the three fixation models; the red label indicates where peak VMS occurred. **a** LPF model; **b** LPF-AOCLS model; **c** LPF-CCLS model

The peak VMS of the implants in the three fixation models occurred at the bending of the locking plate. In the LPF model, the peak VMS of the implant (246.17 mPa) was higher than those in the LPF-AOCLS (232.47 mPa) and LPF-CCLS (231.85 mPa) models (Fig. 7).

### EES-LF

**Fig. 8:**
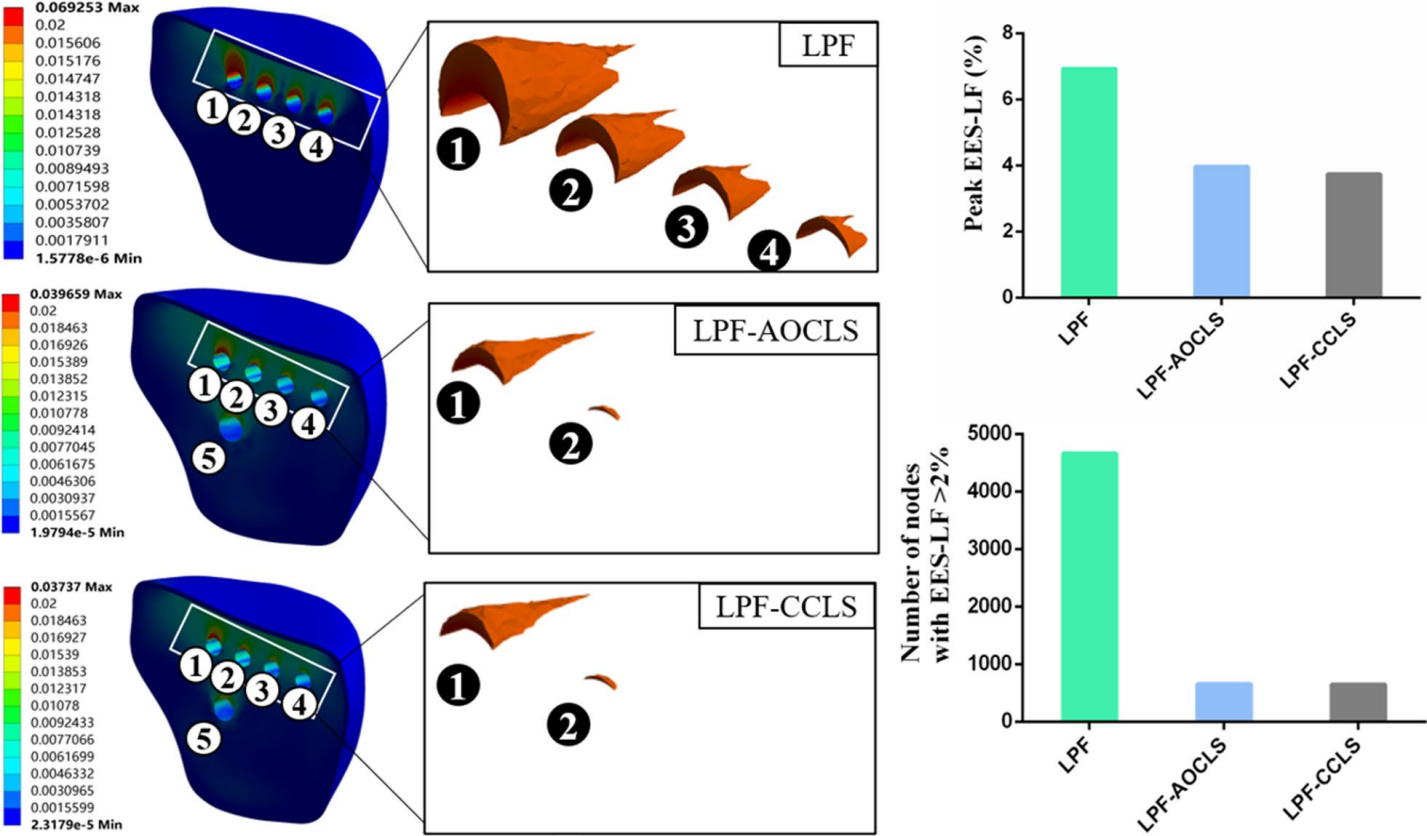
Comparison of peak equivalent elastic strain of the lateral fragment (EES-LF) and nodes with EES-LF > 2%; the numerical indicators depicted in the figure correspond to the screw tunnels in the lateral fragment

In the three fixation models, the peak EES-LF was observed at the posterior screw tunnel close to the fracture plane. The peak EES-LF and nodes with EES-LF > 2% in the LPF model (6.93%, 4660) were higher than those in the LPF-AOCLS (3.97%, 656) and LPF-CCLS (3.74%, 649) models (Fig. 8).

## Discussion

This study investigated the effect of a CCLS on OLTPF stability using an LPF through biomechanical testing and FEA. Biomechanical tests showed that the stability of the OLTPF was enhanced through the IFCF provided by the lag screw. Furthermore, the FEA showed that adding a lag screw reduced the peak VMS of the LP and EES-LF.

Previous studies have shown that the IFCF produced by the lag screws improves the stability of the distal femoral fractures in non-osteoporotic bones [26–28]. However, the role of IFCF in OLTPFs remains unclear. In our study, the effect of IFCF on OLTPF was investigated using biomechanical testing and FEA. The results of the biomechanical tests showed that the LPF-AOCLS and LPF-CCLS groups exhibited significantly higher IAS than that of the LPF group. This was similar to the results of the study by Plecko et al*.* [28] and is significant for patients to bear weight early, restore knee function, and avoid complications related to long-term bed rest. Additionally, we performed a fatigue test consisting of 10,000 loading cycles from 30 to 400 N to simulate a 70-kg adult walking during the 6-week fracture-healing process [29]. No failure in which the MAM-LF was greater than 2 mm was observed in any samples during the loading cycles. However, there were significant differences in MAM-LF in the LPF group compared with those in the LPF-AOCLS and LPF-CCLS groups, indicating that the stability of LPF-AOCLS and LPF-CCLS was superior to that of LPF during the fracture-healing process. In addition, the load-to-failure test demonstrated a significant increase in the failure load in the LPF-AOCLS and LPF-CCLS groups compared to the LPF group. This finding was aligned with existing research on non-osteoporotic bones [19] and supported the hypothesis that the IFCF provided by the lag screw plays a crucial role in enhancing the stability of the LPF in OLTPF.

FEA showed findings similar to those of the biomechanical tests. The results of the MAM-LF in the LPF model were greater than those in the LPF-AOCLS and LPF-CCLS models. This was also reflected in the VMS results. The LPF model exhibited a higher peak VMS for the LP compared to the LPF-AOCLS and LPF-CCLS models. Thus, the IFCF provided by the lag screw was shown to effectively reduce the MAM-LF and load on the LP to protect it from damage. This result agrees with the study by Zhang et al. [19], in which FEA of distal femoral fractures was performed. Moreover, the LPF model exhibited a higher peak EES-LF and a greater number of nodes with EES-LF > 2% compared to the LPF-AOCLS and LPF-CCLS models. This finding implies that the lateral fragment suffered more severe bone damage when fixed using the LPF, suggesting that the IFCF provided by the lag screw could decrease the cutting effect caused by locking screws in practical applications. These results suggest that LP combined with lag screws is a feasible fixation strategy for OLTPFs.

However, the present analysis revealed no significant differences between the LPF-CCLS and LPF-AOCLS groups in the IAS, fatigue test, or load-to-failure test, although the LPF-CCLS group exhibited numerically superior performance compared to the LPF-AOCLS group. There are two possible explanations for this phenomenon. First, although patients with osteoporosis have a low bone density and the trabecular bone structure is too weak to maintain the holding force of the lag screw [30], both AOCLS and CCLS have sufficient holding force in synthetic tibial bones; hence, it was difficult for the AOCLS and CCLS to produce significant differences in the holding force. Second, the limited sample size of the study and slight disparity in IFCF provided by the CCLS and AOCLS may have resulted in a lack of significant differences in the stability of LPF of OLTPF. Based on the biomechanical testing and FEA findings, both CCLS and AOCLS may enhance the stability of the LPF of OLTPF by providing an IFCF.

### Limitations

This study has some limitations. First, only synthetic tibia bones were used, and no experiments were conducted on actual bones. Although this model may not accurately reproduce clinical osteoporotic fracture fixation, it allows for highly reproducible testing using homogeneous material. Second, the force exerted by body weight on the tibia was subject to variation based on the degree of flexion and extension in real life. Accurately replicating these intricate dynamics in the laboratory setting has proven challenging. However, according to published methods, the single test setting used in this study was sufficient to examine fixation stability [31].

## Conclusion

Overall, this analysis showed that both CCLS and AOCLS effectively enhance the stability of OLTPFs using LPF by providing IFCF. Although no significant differences were observed between the CCLS and AOCLS groups, considering the risk of overscrewing in osteoporotic bones, CCLS is recommended for improving the stability of LPF in patients with OLTPF.

## Data Availability

The data that support the findings of this study are available on request from the corresponding author.

## Abbreviations

TPF: Tibial plateau fracture
OLTPF: Osteoporotic lateral tibial plateau fracture
LP: Locking plate
IFCF: Interfragmentary compression force
LPF: Locking plate fixation
CCLS: Combined cancellous lag screw
AOCLS: AO cancellous lag screws
FEA: Finite element analysis
IAS: Initial axial stiffness
MAM-LF: Maximal axial micromotion of the lateral fragment
VMS: von Mises stress
EES-LF: Equivalent elastic strain of the lateral fragment
FE: Finite element

## References

[1] Kosters C, Schliemann B, Raschke MJ (2011). Tibial head fractures in the elderly. Unfallchirurg.

[2] Shen QJ, Zhang JL, Xing GS, Liu ZY, Li EQ, Zhao BC, Zheng YC, Cao Q, Zhang T (2019). Surgical treatment of lateral tibial plateau fractures involving the posterolateral column. Orthop Surg.

[3] Parratte S, Ollivier M, Argenson JN (2018). Primary total knee arthroplasty for acute fracture around the knee. Orthop Traumatol Surg Res.

[4] Honkonen SE (1994). Indications for surgical treatment of tibial condyle fractures. Clin Orthop Relat Res.

[5] Lansinger O, Bergman B, Körner L, Andersson GB (1986). Tibial condylar fractures. a 20-year follow-up. J Bone Joint Surg Am.

[6] Carrera I, Gelber PE, Chary G, Gonzalez-Ballester MA, Monllau JC, Noailly J (2016). Fixation of a split fracture of the lateral tibial plateau with a locking screw plate instead of cannulated screws would allow early weight bearing: a computational exploration. Int Orthop.

[7] Matsunobu T, Maekawa A, Nomoto S, Iwamoto Y (2022). Successful management of radiation-associated insufficiency fracture of the tibial plateau with low-intensity pulsed ultrasound. Am J Case Rep.

[8] Urruela AM, Davidovitch R, Karia R, Khurana S, Egol KA (2013). Results following operative treatment of tibial plateau fractures. J Knee Surg.

[9] Ali AM, El-Shafie M, Willett KM (2002). Failure of fixation of tibial plateau fractures. J Orthop Trauma.

[10] Gardner MJ, Nork SE, Huber P, Krieg JC (2010). Less rigid stable fracture fixation in osteoporotic bone using locked plates with near cortical slots. Injury.

[11] Wang Z, Zheng Z, Wang Y, Zhu Y, Tan Z, Chen W, Hou Z, Zhang Y (2022). Unilateral locking plate versus unilateral locking plate combined with compression bolt for Schatzker I–IV tibial plateau fractures: a comparative study. Int Orthop.

[12] Gao W, Qi X, Zhao K, Feng X, Yang Y, Liu P, Fu D (2023). Lateral locking plate plus antero-posterior lag screws techniques for the management of posterolateral tibial plateau fracture: preliminary clinical results and biomechanical study. Arch Orthop Trauma Surg.

[13] Bel JC (2019). Pitfalls and limits of locking plates. Orthop Traumatol Surg Res.

[14] Barlow JD, Logli AL, Steinmann SP, Sems SA, Cross WW, Yuan BJ, Torchia ME, Sanchez-Sotelo J (2020). Locking plate fixation of proximal humerus fractures in patients older than 60 years continues to be associated with a high complication rate. J Shoulder Elbow Surg.

[15] Xu DQ, Sun PD, Wang J, Yang HL, Liu XJ, Zhao WD (2015). The new shank construct of lag screw improves the maximum compression force for internal fixations: preliminary results. Eur Rev Med Pharmacol Sci.

[16] O’Neill F, Condon F, McGloughlin T, Lenehan B, Coffey C, Walsh M (2012). Validity of synthetic bone as a substitute for osteoporotic cadaveric femoral heads in mechanical testing: a biomechanical study. Bone Joint Res.

[17] Newell N, Rivera TD, Rahman T, Lim S, O’Connell GD, Holsgrove TP (2020). Influence of testing environment and loading rate on intervertebral disc compressive mechanics: an assessment of repeatability at three different laboratories. Jor Spine.

[18] Wilke HJ, Wenger K, Claes L (1998). Testing criteria for spinal implants: recommendations for the standardization of in vitro stability testing of spinal implants. Eur Spine J.

[19] Zhang J, Wei Y, Li G, Wang J, Xu Y (2021). Interfragmentary lag screw and locking plate combination in simple distal femoral fractures: a finite element analysis. Acta Orthop Traumatol Turc.

[20] Lewis GS, Mischler D, Wee H, Reid JS, Varga P (2021). Finite element analysis of fracture fixation. Curr Osteoporos Rep.

[21] MacLeod AR, Simpson AHRW, Pankaj P (2015). Reasons why dynamic compression plates are inferior to locking plates in osteoporotic bone: a finite element explanation. Comput Method Biomec.

[22] Iniguez-Macedo S, Lostado-Lorza R, Escribano-Garcia R, Martinez-Calvo MA (2019). Finite element model updating combined with multi-response optimization for hyper-elastic materials characterization. Materials (Basel).

[23] Zhang L, Yang G, Wu L, Yu B (2010). The biomechanical effects of osteoporosis vertebral augmentation with cancellous bone granules or bone cement on treated and adjacent non-treated vertebral bodies: a finite element evaluation. Clin Biomech (Bristol, Avon).

[24] Bonivtch AR, Bonewald LF, Nicolella DP (2007). Tissue strain amplification at the osteocyte lacuna: a microstructural finite element analysis. J Biomech.

[25] Chen AC, Lin YH, Kuo HN, Yu TC, Sun MT, Lin CL (2013). Design optimisation and experimental evaluation of dorsal double plating fixation for distal radius fracture. Injury.

[26] Perren SM (2002). Evolution of the internal fixation of long bone fractures. The scientific basis of biological internal fixation: choosing a new balance between stability and biology. J Bone Joint Surg Br.

[27] Mardian S, Schmolz W, Schaser KD, Duda GN, Heyland M (2015). Interfragmentary lag screw fixation in locking plate constructs increases stiffness in simple fracture patterns. Clin Biomech (Bristol, Avon).

[28] Mardian S, Schmolz W, Schaser KD, Duda GN, Heyland M (2019). Locking plate constructs benefit from interfragmentary lag screw fixation with decreased shear movements and more predictable fracture gap motion in simple fracture patterns. Clin Biomech (Bristol, Avon).

[29] Plecko M, Lagerpusch N, Pegel B, Andermatt D, Frigg R, Koch R, Sidler M, Kronen P, Klein K, Nuss K, Gedet P, Burki A, Ferguson SJ, Stoeckle U, Auer JA, von Rechenberg B (2012). The influence of different osteosynthesis configurations with locking compression plates (LCP) on stability and fracture healing after an oblique 45° angle osteotomy. Injury.

[30] Rexiti P, Aierken G, Wang S, Abudurexiti T, Abuduwali N, Deng Q, Guo H, Sheng W (2019). Anatomical research on strength of screw track fixation in novel cortical bone trajectory for osteoporosis lumbar spine. Am J Transl Res.

[31] Salduz A, Birisik F, Polat G, Bekler B, Bozdag E, Kilicoglu O (2016). The effect of screw thread length on initial stability of Schatzker type 1 tibial plateau fracture fixation: a biomechanical study. J Orthop Surg Res.

